# A Long-Term Single-Center Study: Motivations and Strategies in Implant Management for Breast Augmentation Revision Surgery

**DOI:** 10.1007/s00266-025-05049-7

**Published:** 2025-07-16

**Authors:** Zhaoyu Chen, Yang Sun, Hui Weng, Jing Tong, Jie Yang, Rongrong Wang, Jiaming Sun

**Affiliations:** 1 Department of Plastic Surgery, Union Hospital, Tongji Medical College, Huazhong University of Science and Technology, 1277 Jiefang Avenue, Wuhan, 430022 China; 2 Wuhan Clinical Research Center for Superficial Organ Reconstruction, 1277 Jiefang Avenue, Wuhan, 430022 China; 3 Department of Medical Records Management and Statistics, Union Hospital, Tongji Medical College, Huazhong University of Science and Technology, Wuhan, 430022 China

**Keywords:** Breast augmentation, Revision surgery, Implant removal, Implant replacement

## Abstract

**Background:**

Revision surgery after breast augmentation is performed for various reasons. Understanding the causes and patients’ attitudes toward implant management (removal or replacement) is critical for improving outcomes.

**Methods:**

A retrospective review of medical records from 2015 to 2023 was conducted, categorizing patients by the reasons for revision surgery and implant management approach. Demographic information, implant duration, and postoperative satisfaction were collected, followed by statistical analysis of trends and outcomes.

**Results:**

A total of 133 patients (mean age 43.1) were included: 93 in the medical, 32 in the aesthetic, and 8 in the psychological groups. Of these, 94 opted for implant removal, and 39 chose replacement. Reasons for revision did not significantly influence implant management decisions. No significant differences were found in age, implant duration, or BMI across groups, whether categorized by reason for revision surgery or implant management. Common issues in the medical group included capsular contracture and implant rupture, while the aesthetic group cited shape dissatisfaction. Over 80% of patients reported high satisfaction with post-revision outcomes.

**Conclusions:**

Implant management decisions were not significantly affected by revision reasons or demographic factors. While medical complications primarily drive revision surgery, many patients seek surgery for subjective reasons. These findings highlight the need for comprehensive patient education, psychological support, and personalized surgical planning, with both implant removal and replacement achieving high satisfaction.

**Level of Evidence III:**

This journal requires that authors assign a level of evidence to each article. For a full description of these Evidence-Based Medicine ratings, please refer to the Table of Contents or the online Instructions to Authors www.springer.com/00266.

## Introduction

Breast augmentation is one of the most popular cosmetic surgeries worldwide, and silicone implants are widely used to improve breast aesthetics. Despite advancements in implant design, such as cohesive gel implants, and surgical techniques, a significant proportion of women eventually undergo implant revision surgery, including removal or replacement. Notably, revision surgery has shown the most significant growth within the breast procedures group since 2015 [1]. Patients decided to have breast implants revision surgery for various reasons, including implant-related complications, health concerns, and changes in personal preferences. [2–5]

Several studies have indicated that approximately 20% of women experience procedure-related complications following primary breast augmentation, including implant displacement, rippling, capsular contracture, and late fluid accumulation such as hematomas or seromas. Meanwhile, patient satisfaction is generally high after implant removal, with improved symptoms [5–8]. Furthermore, online resources are crucial in influencing patients’ decisions to undergo breast implant revision surgery. They provide easy access to information on potential risks, complications, and alternatives to implants, raising awareness of conditions such as capsular contracture and breast implant illness (BII). Peer experiences and testimonials on social media and forums provide emotional support and validation, encouraging patients to consider removal. However, redundancy associated with information acquisition presents the disadvantage of potential inaccuracies, as patients may encounter misinformation or exaggerated risks, highlighting the importance of consulting medical professionals in making informed decisions.

Despite the increasing number of patients seeking breast implant revision surgery, there remains a paucity of data regarding the long-term trends, underlying motivations, and management strategies associated with these procedures. Gaining insights into these factors is essential for informing surgeons’ perioperative practices, standardizing procedural protocols, and improving patient education and counseling efforts.

This study analyzes a nine-year single-center experience with breast implant revision surgeries, focusing on two key aspects: the motivations for undergoing revision surgery and the management of breast implants during these procedures, specifically regarding removal and replacement strategies. Additionally, the study examines the relationship between these factors and various patient parameters, such as the duration of implant placement, age at the time of primary augmentation, and age at the time of revision surgery. Patient satisfaction was also assessed through follow-up to better understand patient preferences and decision-making. By addressing these dimensions, this study aims to deepen the understanding of patient needs and inform strategies for optimizing patient education and personalized treatment approaches.

## Material and Methods

This retrospective study examined patients treated at the Department of Plastic Surgery of Union Hospital, affiliated with Tongji Medical College, Huazhong University of Science and Technology, from 2015 to 2023. The study cohort consisted exclusively of individuals who underwent breast augmentation for aesthetic purposes, excluding those who underwent reconstruction surgeries due to breast absence. Patients were categorized into three distinct groups according to the primary motivations for undergoing revision surgery: the medical group, characterized by clear symptoms of complications; the aesthetic group, which involved dissatisfaction with appearance; and the psychological group, motivated by psychological factors. Alternatively, patients were also classified into two groups based on implant management: those undergoing implant removal and those opting for implant replacement. The analysis focused on the relationship between the duration of implant placement, the age at primary breast augmentation, the age at the time of revision surgery, and the occurrence of complications across these groups. Additionally, the study considered fundamental demographic characteristics of the patients and explored the correlation between patients’ decisions regarding implant management and a range of influencing factors.

A Likert satisfaction scale was used for the follow-up assessment, wherein patients were instructed to evaluate their experiences on a scale ranging from 1 to 5, (1 = very dissatisfied; 2 = dissatisfied; 3 = neutral; 4 = satisfied; and 5 = very satisfied). The patients were asked to respond to the following questions: 1. To what extent are you satisfied with the outcomes of your prior breast augmentation surgery? 2. How would you assess your satisfaction following the revision surgery performed at our department? 3. Please provide separate ratings for your satisfaction with the overall appearance, shape, and softness of your breasts.

Data were collected from the electronic medical records of patients at Union Hospital after approval from the Institutional Review Board. Descriptive analysis was performed to assess the characteristics of the sample. The analysis included the calculation of the mean, standard deviations, and ranges for continuous variables. Absolute and relative frequencies were determined for categorical variables. Statistical analyses were performed using IBM SPSS Statistics (version 27), which included one-way ANOVA and t tests for comparing continuous variables and Chi-square tests for categorical variables. Statistical significance was set at *p* < 0.05.

## Results

**Fig. 1:**
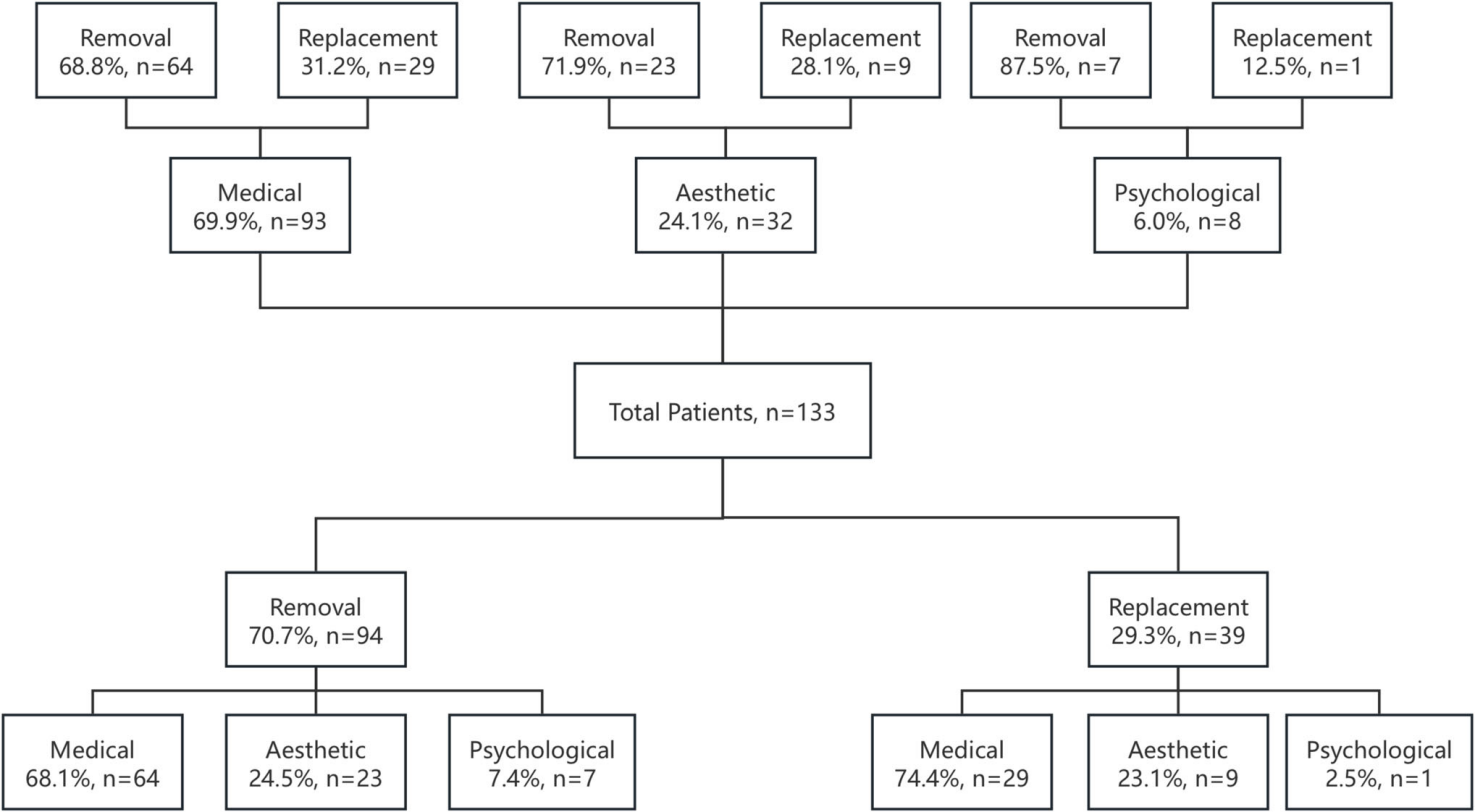
Overview of all patients based on the purpose and type of their revision surgeries

Between 2015 and 2023, a total of 133 eligible patients (mean age of 43.1 years; 95% CI range was 41.3–44.9 years) were enrolled in the study. The patients were divided into three groups: 93 (69.9%) in the medical group, 32 (24.1%) in the aesthetic group, and eight (6.0%) in the psychological group. Among these patients, 94 (70.7%) underwent complete implant removal, while 39 (29.3%) opted for implant replacement (Fig. 1). Statistical analysis revealed no significant differences in the age at which breast augmentation was first performed, age at the visit, or BMI values across the groups. During the study period, 374 patients underwent breast-related procedures at our institution, including 241 (64.4%) who received primary augmentation for aesthetic purposes and 133 (35.6%) who underwent revision surgery. Among the 133 patients included in this revision cohort, 18 had received their initial augmentation at our institution, while 115 had undergone primary surgery at external clinics. The duration of implant placement varied among the three groups, with an overall average of 9.9 years. No statistically significant differences were observed in the duration of the implant placement. Seven (5.2%) patients required more than one revision procedure, most of which occurred more than five years after the initial procedure (Table 1).

**Table 1:** Demographics and clinical characteristics

		Medical		Aesthetic		Psychological	
		Removal	Replacement	Removal	Replacement	Removal	Replacement
No. of patients		64	29	23	9	7	1
Age, year	Primary	33.48 ± 8.50	30.86 ± 6.17	33.39 ± 8.78	32.06 ± 9.21	31.57 ± 3.16	30
	Now	44.19 ± 10.18	41.97 ± 9.39	41.96 ± 12.52	40.89 ± 8.64	42.57 ± 9.95	55
BMI, kg/m2		21.17 ± 2.61	20.39 ± 2.45	20.25 ± 2.25	19.78 ± 1.63	20.71 ± 0.90	20
Primary surgery	In house	11	2	2	0	2	1
	Other clinic	53	27	21	9	5	0
Interval, year		9.95 ± 7.71	10.73 ± 8.66	8.53 ± 7.07	7.72 ± 3.33	11.00 ± 9.49	25

**Fig. 2:**
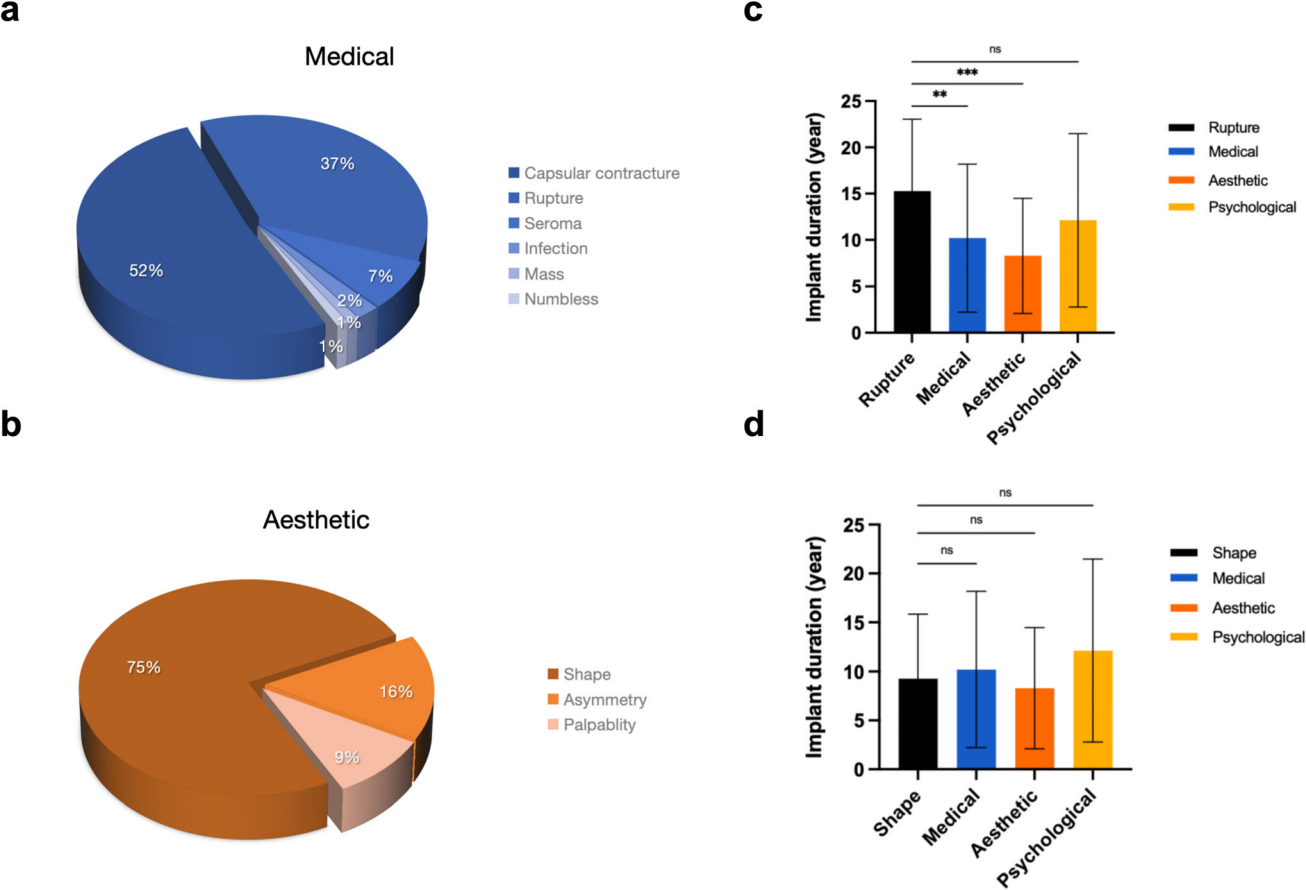
(**a**, **b**) Reasons for revision surgery in medical and aesthetic groups. (**c**, **d**) Analysis of implant placement duration in individuals with implant rupture and those dissatisfied with breast shape with the three cohorts in this study. (**p < 0.01, ***p < 0.001, and ns stands for not significant)

The three patient cohorts demonstrated significant differences in their motivations for undergoing revision surgery. Capsular contracture (52%, 48/93) was the most prevalent concern in the medical group, followed by implant rupture (37%, 34/93), with 7% (7/93) of the patients presenting for intervention primarily due to seroma (Fig. 2a). Most patients in the aesthetic group sought treatment because of dissatisfaction with their shape (75%, 24/32), with 16% (5/32) reporting bilateral breast asymmetry and 9% (3/32) pursuing additional intervention due to palpability (Fig. 2b). In the psychological group, all patients sought medical attention because of anxiety about the implants. Figure 2c illustrates that the duration of implant placement in the cohort undergoing surgical intervention for rupture was significantly higher than that observed in the medical and aesthetic groups (15.3, 95% CI: 12.6-18.0, *p* < 0.01). Nevertheless, this apparent intergroup disparity was not observed within the primary complication cohort of the aesthetic group (Fig. 2d).

**Fig. 3:**
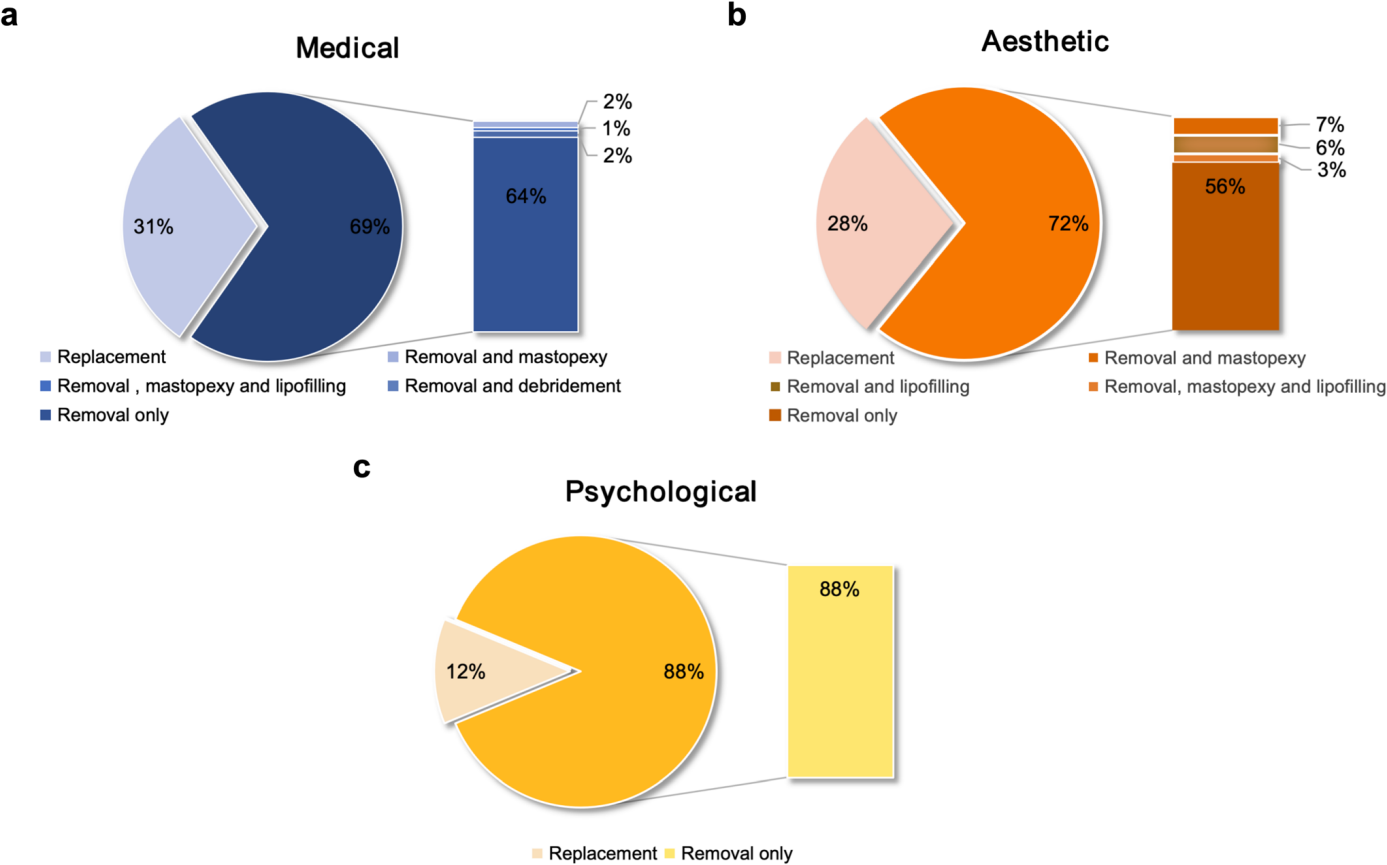
Overview of revision surgery across the medical (**a**), aesthetic (**b**), and psychological group (**c**)

As demonstrated in Fig. 3, the majority of patients did not opt for implant replacement during revision surgery, whether across all patients (70.7%, 94/133) or within each cohort. In the medical group, 64% of patients chose implant removal without additional procedures, while 5% required further interventions after removal (Fig. 3a). In the aesthetic group, 72% opted for implant removal alone, while 16% underwent additional surgeries post-removal (Fig. 3b). In the psychological group, none of the patients who chose implant removal required subsequent interventions (Fig. 3c). The proportions of implant replacement in the three groups were 31%, 28%, and 12%, respectively. Overall, additional surgical steps are common in patients selected for implant removal. In these surgical procedures, emphasis is placed on techniques that can effectively attain desired outcomes while facilitating ease of execution.

**Fig. 4:**
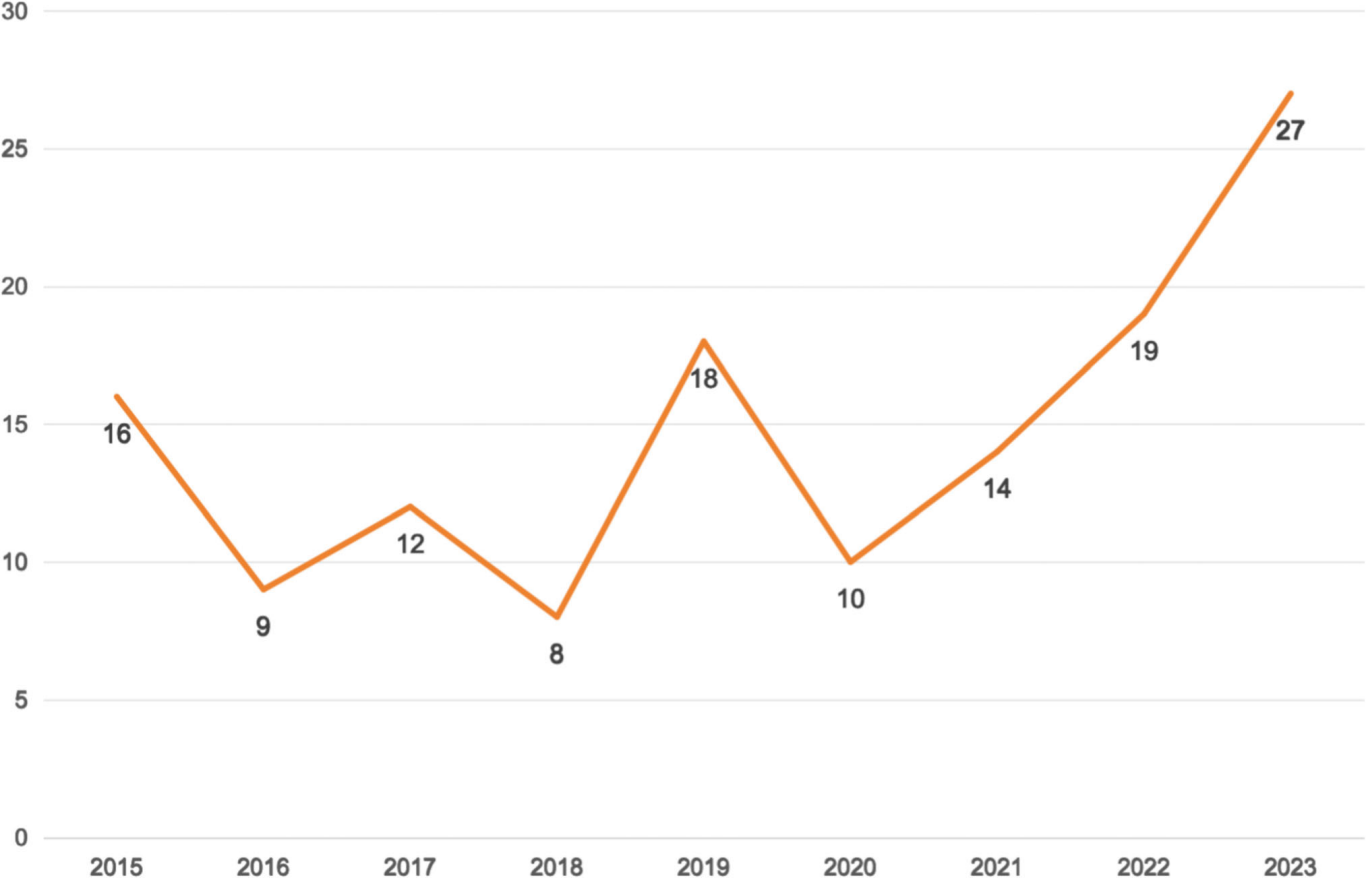
Distribution of patients requesting breast implant removal during the study period

**Fig. 5:**
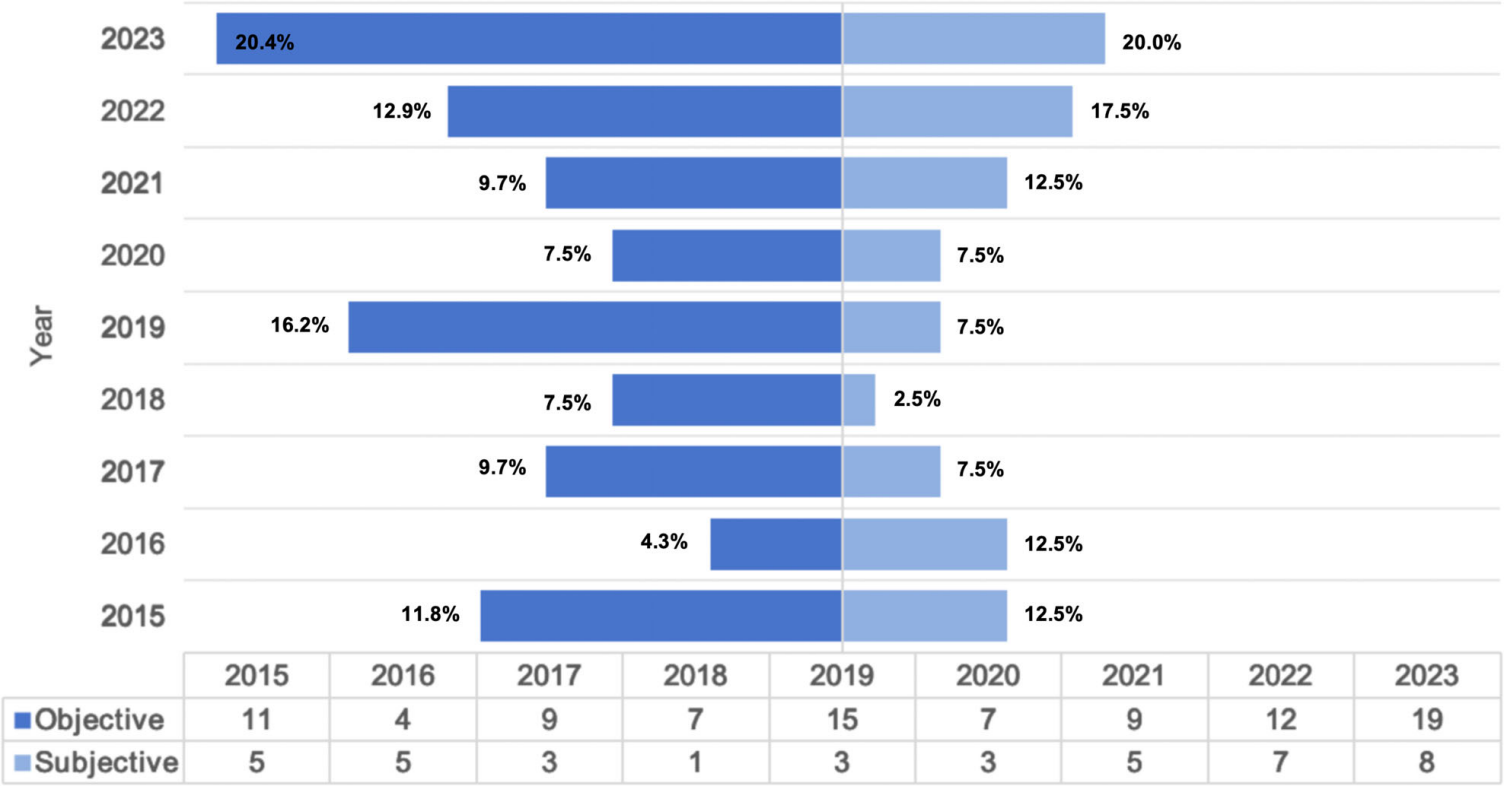
Distribution of patients undergoing implant removal surgery over the study period due to objective or subjective factors

Figure 4 illustrates the gradual increase in the trend of breast implant revision surgery. The reasons patients provide for undergoing this procedure can be classified into two categories: objective reasons, which are based on medical groups, and subjective reasons, which encompass aesthetic and psychological groups. The primary rationale was consistently represented by objective factors for each year, demonstrating a general upward trend over time (Fig. 5).

**Fig. 6:**
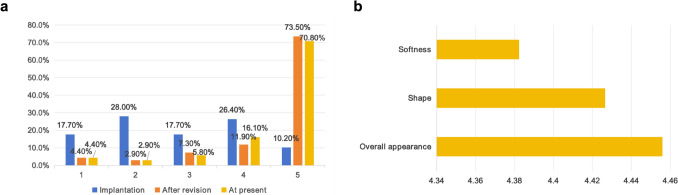
**a** Patient satisfaction with implant experiences measured at three perioperative time points. **b** Mean overall patient satisfaction across different aspects after surgery, based on Likert scale ratings

Figure 6a summarizes the patient satisfaction responses recorded immediately after implantation, after revision surgery, and at present. Over 80% of patients indicated that they were satisfied or very satisfied following implant removal, and this elevated level of satisfaction has persisted to the present time. Furthermore, the patient exhibited a high degree of satisfaction regarding the softness, shape, and overall aesthetic appearance of the breast (Fig. 6b).

All key statistical results are outlined in Table 2.

**Table 2:** Outline of key statistical findings from the study

Longer mean duration of implant placement in rupture patients compared to other three cohorts
Reason for revision surgery was not associated with the choice of implant removal or replacement
Choice between implant removal and replacement was not associated with the type of complication
Choice of implant removal or replacement was unrelated to age at augmentation, age at revision, or implant duration
Reason for revision surgery was not associated with the institution performing the primary augmentation surgery.
Reason for revision surgery was not associated with age at primary augmentation, age at revision surgery, or implant duration
Degree of satisfaction was not associated with the choice of implant removal or replacement

## Discussion

Although modern silicone implants are considered safe and effective, with high patient satisfaction rates and low complication risks, breast implants revision following augmentation procedures is becoming an increasingly prevalent practice [9, 10]. The data obtained from this research indicate that, excluding the effects of the COVID-19 pandemic, there has been a consistent annual increase in the demand for breast implant revision surgery (Fig. 4). This trend is also observed in other countries, potentially because of concerns about the BII and increased public awareness of the potential systemic effects [11, 12]. Therefore, it is imperative to understand the underlying motivations for these decisions and the current trends in decision-making, as this knowledge is vital for effectively advising patients regarding their preoperative surgical options and management.

Following the exclusion of patients who underwent breast augmentation surgery for reconstructive purposes after breast tumor excision, 133 patients were classified into three distinct groups according to their motivations for revision: medical, aesthetic, and psychological. The decisions regarding revision surgery and implant management were not associated with the age at the time of the initial breast augmentation, the age at the time of revision surgery, or the duration of implant placement. This lack of variation suggests that the sample is homogeneous, thereby supporting the validity of future research. A total of 13.5% of patients underwent their prior breast augmentation procedures in our department. This observation aligns with the perspective proposed by Forster [13], suggesting that patients who experience complications or dissatisfaction with the aesthetic outcomes of surgery are more likely to seek revision procedures from providers other than the original operating surgeon.

The primary concern of the medical group was the objective complications associated with physical symptoms, with capsular contracture accounting for 52% of the cases among the patient population. This finding is basically consistent with prior studies, which report the incidence of capsular contracture ranging from 10.6% to 45%, identifying it as the most common indication for revision surgery [13–16]. Established risk factors for capsular contracture include the use of smooth-surfaced implants, subglandular placement, and extended postoperative follow-up periods. In our practice, total capsulectomy is the preferred approach for managing capsular contracture, as it is often considered necessary to address both clinical symptoms and anatomical distortion. [17, 18]

Silicone gel breast implants have a limited lifespan, and their rupture rates increase over time. Studies have indicated that 30%–70% of implants may rupture or leak after 5–10 years [19–21]. The integrity of implants is not associated with capsular contracture, but reconstruction cases exhibit higher contracture rates than cosmetic cases [22]. Clinical follow-up alone is insufficient for detecting ruptures, as most were identified incidentally during imaging. Regular imaging monitoring is recommended, particularly after 4–7 years [20, 23]. In the present study, the average duration of implant placement among patients with rupture was 15.3 years. This finding highlights the critical need to counsel patients about the limited longevity of implants and the importance of regular postoperative surveillance. In addition, timely imaging evaluation should be conducted even in asymptomatic individuals, especially as they approach the expected lifespan of the implant, to facilitate early detection and appropriate intervention.

A seroma is considered "late" if it manifests more than one year after implantation. Approximately 1%–2% of breast implant placements result in late seroma, most of which are associated with textured implants. Although plastic surgeons routinely screen for BIA-ALCL when late periprosthetic fluid collections are identified, most late seromas are caused by conditions and capsular abnormalities unrelated to BIA-ALCL [18, 24]. In this study, seven patients presented for medical evaluation primarily due to seroma, and the pathological findings subsequently excluded the possibility of malignant lesions.

Patients who pursue treatment because of dissatisfaction with the appearance of their breasts may experience a shift in aesthetic preferences over time. Although surgical intervention may initially align with their aesthetic preferences, their subsequent expectations regarding appearance may evolve. Conversely, patients who express a desire for larger breast sizes may initially encounter restrictions on the use of larger silicone implants owing to constraints related to tissue capacity. However, after implant expansion, these patients may seek consultation to discuss possibly replacing their implants with larger-capacity silicone options. The interpretation of this finding must be considered speculative.

Breast augmentation techniques significantly affect complication rates and outcomes. Inframammary incisions and subglandular placement are associated with lower complication risks, while implants > 350 mL increase the risk. Submuscular placement reduced the risk of capsular contracture [25]. Shaped textured gel implants and subpectoral pockets demonstrated lower complication rates [26, 27]. Due to significant heterogeneity in primary augmentation procedures across different institutions and surgeons, the total number of augmentation cases was not included in this study. A well-defined process, including patient education, tissue-based planning, refined surgical technique, and structured postoperative management, can improve the outcomes.

Psychological outcomes following breast augmentation are multifaceted. While most patients report improved body image, self-esteem, and overall satisfaction [22, 28], underlying psychopathology, including body dysmorphic disorder, is not uncommon [29]. Some studies have linked breast implants to elevated risks of anxiety, depression, and even suicide [30, 31]. Personality traits, particularly neuroticism, may predispose individuals to BII, which is characterized by heightened somatic symptoms, depression, anxiety, and health-related anxiety [32, 33]. BII is often described as distressing and debilitating, affecting multiple aspects of life [34]. Although a pilot study using fMRI found no evidence of brain alterations in BII patients, it did confirm significantly worse psychosocial symptoms compared to controls [32]. Studies have also shown that explantation of silicone breast implants can lead to significant improvement or resolution of BII-related symptoms [35–38]. In our study, 88% of patients admitted for psychological reasons chose implant removal alone (Fig. 3). Following implant removal, their symptoms improved, and patient satisfaction was high. These findings underscore the significance of comprehensive psychological assessment and patient management before and after surgery. This highlights the need for close collaboration between mental health specialists and plastic surgeons to ensure optimal outcomes and patient safety.

Benito-Ruiz [8] reported that simple implant removal can yield favorable aesthetic outcomes and high patient satisfaction, influenced by factors such as capsular contracture, revision timing, and payment method. Optimal results may be achieved through individualized surgical planning, including adjunct procedures like mastopexy in appropriate candidates [2, 5]. In our study, patient satisfaction following revision surgery was consistently high and stable over time. While most patients were content with implant removal, a subset expressed interest in further procedures, primarily due to concerns about breast volume loss. This perceived "emptiness" prompted consideration of re-augmentation or alternative strategies such as mastopexy or lipofilling, which are increasingly recognized as effective options to improve contour and restore volume.

A limitation of this study is the lack of complete data on implant surface types. Since only a minority of patients received their primary augmentation at our institution, and implant details were often unavailable due to rupture or unmarked posterior surfaces, analysis of surface characteristics and their potential impact on outcomes could not be reliably conducted.

## Conclusions

Implant management decisions are shaped not only by medical complications but also by evolving aesthetic preferences and psychological factors. While clinical indications remain central, a substantial number of patients seek revision for nonmedical reasons, underscoring the importance of comprehensive preoperative education, timely follow-up, and psychological support. Surgeons play a pivotal role in guiding patients toward informed decisions, whether to remove or replace implants, by aligning expectations with achievable outcomes and clearly communicating the limitations of revision procedures.
